# Identification of the ISWI Chromatin Remodeling Complex of the Early Branching Eukaryote Trypanosoma brucei*

**DOI:** 10.1074/jbc.M115.679019

**Published:** 2015-09-15

**Authors:** Tara Stanne, Mani Shankar Narayanan, Sophie Ridewood, Alexandra Ling, Kathrin Witmer, Manish Kushwaha, Simone Wiesler, Bill Wickstead, Jennifer Wood, Gloria Rudenko

**Affiliations:** From the ‡ Division of Cell and Molecular Biology, Department of Life Sciences, Sir Alexander Fleming Building, Imperial College London, South Kensington, London SW7 2AZ, United Kingdom and; the § School of Life Sciences, University of Nottingham, Nottingham NG7 2UH, United Kingdom

**Keywords:** chromatin remodeling, nucleosome, RNA polymerase I, transcription, Trypanosoma brucei, ISWI, VSG expression site

## Abstract

ISWI chromatin remodelers are highly conserved in eukaryotes and are important for the assembly and spacing of nucleosomes, thereby controlling transcription initiation and elongation. ISWI is typically associated with different subunits, forming specialized complexes with discrete functions. In the unicellular parasite *Trypanosoma brucei*, which causes African sleeping sickness, TbISWI down-regulates RNA polymerase I (Pol I)-transcribed variant surface glycoprotein (VSG) gene expression sites (ESs), which are monoallelically expressed. Here, we use tandem affinity purification to determine the interacting partners of TbISWI. We identify three proteins that do not show significant homology with known ISWI-associated partners. Surprisingly, one of these is nucleoplasmin-like protein (NLP), which we had previously shown to play a role in ES control. In addition, we identify two novel ISWI partners, regulator of chromosome condensation 1-like protein (RCCP) and phenylalanine/tyrosine-rich protein (FYRP), both containing protein motifs typically found on chromatin proteins. Knockdown of RCCP or FYRP in bloodstream form *T. brucei* results in derepression of silent variant surface glycoprotein ESs, as had previously been shown for TbISWI and NLP. All four proteins are expressed and interact with each other in both major life cycle stages and show similar distributions at Pol I-transcribed loci. They are also found at Pol II strand switch regions as determined with ChIP. ISWI, NLP, RCCP, and FYRP therefore appear to form a single major ISWI complex in *T. brucei* (TbIC). This reduced complexity of ISWI regulation and the presence of novel ISWI partners highlights the early divergence of trypanosomes in evolution.

## Teaser

**Background:** Eukaryotes typically encode a range of ISWI chromatin remodeling complexes with different functions.

**Results:** We have identified and analyzed three novel ISWI partners in the early branching eukaryote *Trypanosoma brucei*.

**Conclusion:** *T. brucei* appears to have a single major ISWI complex.

**Significance:** This unusually simple ISWI configuration could be a consequence of the relative lack of transcriptional regulation in this ancient eukaryote.

## Introduction

Eukaryotes package their genomic DNA into chromatin, whereby DNA is wrapped around octamers of histones forming nucleosomes. This allows the compaction of extensive stretches of DNA into the restricted space of the nucleus as well as being a major factor in controlling DNA access. For example, the exact phasing or degree of compaction of nucleosomes can either block or expose promoter sequences to recognition by the transcriptional machinery (1, 2). Chromatin remodeling therefore plays a major role in the regulation of gene expression, in addition to a range of other processes, including chromosome segregation and DNA replication and repair (3–6).

Chromatin remodelers in the ISWI family are highly conserved among eukaryotes and play a critical role in nucleosome assembly and spacing as well as in the organization of chromatin at a higher level in the cell (7–10). ISWI has a highly conserved SWI2/SNF2 family ATPase domain, which provides the motor for chromatin remodeling, and characteristic HAND-SANT-SLIDE domains with DNA binding activity (3, 5). Using DNA-dependent ATPase activity, ISWI remodelers change nucleosome spacing to promote chromatin assembly, which often results in the repression of transcription (11, 12). In addition to their role in remodeling existing nucleosomes, they can also facilitate the *de novo* assembly of nucleosomes in concert with core histone chaperones (13).

ISWI invariably functions as part of a complex, and different eukaryotes have a diverse array of ISWI complexes, each with a discrete function (8). It is becoming increasingly clear that the ISWI partner subunits have a regulatory role and determine ISWI complex function (8, 10). In *Saccharomyces cerevisiae*, there are two different ISWI variants (Isw1 and Isw2), which in combination with different subunits, form a total of four different complexes (10). ISWI (Isw1) together with the Ioc3 subunit forms the Isw1a complex, which binds Pol^4^ II promoters and excludes the basal Pol II transcription machinery, thereby preventing transcription initiation (14). In contrast, Isw1 partnered with the Ioc2 and Ioc4 subunits forms the Isw1b complex, which regulates Pol II transcription elongation and termination (15–17).

In *Drosophila melanogaster*, six different functional ISWI complexes have been identified (CHRAC, ACF, NURF, RSF, ToRC, and NoRC), each containing ISWI bound to various combinations of nine different subunits (10, 18). Among these, the CHRAC and ACF complexes appear to have general roles in facilitating nucleosome sliding (19, 20). NURF appears to be particularly important for the epigenetic regulation of stem cells within the testis (21). RSF has a role in assembly of chromatin through the replacement of histone variants in addition to chromatin remodeling activities (22). ToRC is involved in the regulation of Pol II transcription (23), whereas NoRC is a nucleolar chromatin remodeling factor involved in silencing Pol I-mediated transcription of the rDNA repeats (24).

In mammalian cells (where the ISWI equivalents are referred to as SNF2H or SNF2L/SMARCA1), at least seven different ISWI complexes have a similar broad range of functions, including facilitating DNA repair (25, 26), activating Pol III transcription (27), or playing a role in the differentiation of somatic cells (28). Similar to in *Drosophila*, an NoRC complex is also present, which mediates the epigenetic regulation of rRNA genes as well as heterochromatin formation at repetitive regions, including the telomeres and centromeres (29–31).

The African trypanosome *Trypanosoma brucei* is a unicellular eukaryote and causative agent of African sleeping sickness (32). Trypanosomes are evolutionarily separated from eukaryotic model organisms and are in a different eukaryotic supergroup (Excavata) from animals and fungi (Opisthokonta) (33). As a consequence, *T. brucei* has unexpected features, including the organization of its genome. Unusually, trypanosome chromosomes consist predominantly of extensive polycistronic transcription units, which are constitutively transcribed by RNA Pol II (34–36). There is no evidence for regulated Pol II transcription in *T. brucei.* Levels of Pol II-derived transcripts are controlled post-transcriptionally through a variety of mechanisms, including co-transcriptional RNA degradation as well as RNA stability elements (37, 38).

Another unusual feature is that RNA Pol I transcribes a subset of protein-coding genes in addition to the rDNA (39). These include the genes encoding the variant surface glycoprotein (VSG), which forms an essential protective coat on the bloodstream form trypanosome (40, 41). Although an individual trypanosome can have a repertoire of more than 2000 *VSG* genes (42, 43), only one *VSG* is transcribed at a time from one of about 15 telomeric *VSG* expression sites (ESs) (44, 45). The molecular mechanisms behind this monoallelic control of *VSG* ESs still remain to be elucidated.

What is the role of chromatin in an organism that has little transcriptional control and does not regulate Pol II transcription units? First of all, chromatin proteins are likely to be important for Pol II transcription in *T. brucei.* Putative Pol II transcription initiation sites have a simple structure lacking canonical Pol II promoter elements (35). No defined motifs for Pol II promoters have yet been identified; however, the H4K10ac acetylation and H3K4me3 histone modifications and H2AZ and H2BV histone variants are enriched at the probable sites of transcription initiation (35, 46). It is therefore likely that these epigenetic marks play an important role in defining a functional Pol II promoter.

In addition, it is now clear that chromatin remodeling plays a key role in the control of *VSG* ESs. The active *VSG* ES is highly depleted of nucleosomes compared with the silent ESs (47, 48). In addition, a steadily increasing number of chromatin proteins, chromatin remodelers, and histone modifiers have now been shown to impact *VSG* ES transcriptional control (49–52).

The first chromatin remodeler discovered to play a role in *VSG* ES regulation is TbISWI (53). Knockdown of TbISWI results in 30–60-fold derepression of a reporter inserted immediately downstream of a silent ES promoter as well as transcriptional read-through in the silent telomeric ESs extending to the telomeric *VSG* genes (53, 54). In addition to the role of TbISWI in silencing *VSG* ESs, TbISWI was also found to be enriched at transcriptional strand switch regions (SSRs) containing Pol II promoters and terminators (35, 54). Because ISWI is invariably part of different functional complexes in other eukaryotes, we attempted to elucidate the role of ISWI complex(es) in *T. brucei*.

Here we identify and analyze three novel ISWI partners in *T. brucei* that are expressed in both the bloodstream form (BF) and the procyclic form (PF) present in the tsetse fly insect vector. Surprisingly, these ISWI-interacting proteins include the nucleoplasmin-like protein (NLP), which we have previously shown to have a similar role to TbISWI in down-regulating ESs (55). We also identify two previously uncharacterized proteins: RCCP and FYRP. All of our experimental evidence points to the presence of a single major ISWI complex in *T. brucei*, although we cannot rule out the presence of minor subcomplexes. This relatively simple configuration of ISWI could be a consequence of the relative lack of extensive transcriptional control in this primitive eukaryote.

## Experimental Procedures

### 

#### 

##### Trypanosome Strains and Culturing

PF *T. brucei brucei* 427 was maintained at 27 °C in SDM-79 medium supplemented with 10% heat inactivated fetal calf serum and 5 mg ml^−1^ hemein (56). BF *T. brucei* 427 was cultured at 37 °C in HMI-9 medium supplemented with 15% fetal calf serum (57).

For tandem affinity purification (TAP), TbISWI (GeneDB: Tb927.2.1810) and NLP (GeneDB: Tb927.10.5450) were tagged at the C terminus with a Protein C-tobacco etch virus (TEV) protease site-Protein A (PTP) epitope (58) in PF *T. brucei* 427. In order to ensure functionality of the TbISWI-PTP protein, PF lines were generated where the second TbISWI allele was knocked out using the pSpot5KOPhleo construct (54). Similarly, the NLP-PTP protein was shown to be functional through generation of cell lines where the second NLP allele was knocked out using the pBSphleoNLPKO construct (55).

For the co-immunoprecipitation experiments, proteins were tagged *in situ* at the endogenous locus at the C terminus using either a triple Myc epitope or a triple HA epitope using either the pMoTAG42M or pMoTAG4H construct (59). These constructs were transfected into wild type PF cells or the BF 221GP1(VO2+) line (60). This line has an active *VSGVO2* expression site, which is maintained using G418 selection, and the *eGFP* and puromycin resistance genes in the silent *VSG221* expression site.

For the expression site derepression and growth rate studies, the BF *T. brucei* T3-SM cell line was used (53). This cell line is a derivative of the “single marker” cell line (61) and contains an active *VSGT3* expression site maintained with blasticidin selection and silent *eGFP* and *VSG221* genes in the inactive *VSG221* expression site. RNAi constructs to knock down either RCCP (GeneDB: Tb927.11.10330) or FYRP (GeneDB: Tb927.7.1060) were integrated into the *T. brucei* minichromosomes to generate either *T. brucei* T3-RCCP or *T. brucei* T3-FYRP. The BF *T. brucei* 90-13 cell line (61) was used for the introduction of both epitope-tagged proteins and RNAi constructs. RNAi was induced with tetracycline to monitor for knockdown of FYRP in the FYRP-HA epitope-tagged line.

##### DNA Constructs

TbISWI was tagged at the C terminus with the PTP epitope for TAP using the pC-PTP construct as described previously (58). The last 899 bp of the TbISWI C terminus was amplified and cloned into the pC-PTP-hygro vector. This was digested with AvaI and integrated via a single crossover. The final 687 bp of the NLP C terminus and positions 68–790 of the NLP downstream sequence were amplified and cloned into the pC-PTP-hygro vector. This was digested with ApaI and SacI and integrated via a double crossover. TbISWI and NLP were epitope-tagged with the triple Myc and/or HA epitopes as described previously (54, 55). A 490-bp fragment (positions 1449–1938) of the RCCP open reading frame (ORF) was amplified, and a 500-bp fragment of the 3′ downstream region (positions 1–500) was inserted into the pMOTag4H or pMOT43MB vector. A 494-bp fragment (positions 989–1173) of the FYRP ORF was amplified, and a 370-bp fragment (positions 1–370) of the 3′ downstream region were inserted into the pMOTag4H or pMOTag43MB vector. The p2T7-177_hygro construct targeted to minichromosomes was used for RNA interference experiments (62). A 485-bp fragment (positions 319–803) of the RCCP ORF and a 477-bp fragment (positions 697–1173) of the FYRP ORF were inserted between the opposing T7 promoters of this construct.

##### Tandem Affinity Purification

The C termini of TbISWI and NLP were tagged with the PTP epitope (58), and TAP was performed as described (63). Briefly, approximately 2 liters of PF *T. brucei* expressing PTP epitope-tagged TbISWI or NLP were lysed by Dounce homogenization and shock-frozen. Proteins were extracted on ice for 20 min and the lysates were centrifuged twice at 20,000 × *g* at 2 °C. The supernatant was incubated at 4 °C with equilibrated IgG-Sepharose Fast Flow bead suspension (GE Healthcare) in a Poly-Prep chromatography column (Bio-Rad) with protease inhibitors for 3.5 h. The beads were washed, and the Protein A portion of the tag was removed by the addition of AcTEV protease (Invitrogen). The TEV eluate was incubated with anti-Protein C matrix (Roche Applied Science) in a fresh Poly-Prep column overnight at 4 °C. The anti-Protein C matrix was washed, and the final TAP-purified material was eluted with EGTA. The purified product was concentrated using a vacuum concentrator and StrataClean resin (Stratagene) before separation under denaturing or nondenaturing conditions on 4–15% SDS-polyacrylamide or polyacrylamide gels (Bio-Rad). Bands were visualized with Imperial Protein Stain (Thermo Scientific) or Silver Stain (Thermo Scientific), excised as specified, and subjected to analysis by liquid chromatography-tandem mass spectrometry (Central Proteomics Facility, University of Oxford).

##### Co-immunoprecipitation

For co-immunoprecipitation (co-IP) analysis, TbISWI, NLP, RCCP, and FYRP were tagged at the C terminus with either a triple Myc epitope using the pMoTAG43M vector (59) or with a triple HA epitope using the pMoTAG4H vector (54, 55, 59) in PF and BF cell lines. Cell extracts were prepared as for the TAP tagging protocol (63), except that 0.1% Nonidet P-40 was added while extracting protein. Sepharose CL-4B columns (GE Healthcare) were prepared with ice-cold IP buffer (150 mm sucrose, 20 mm l-glutamic acid, 20 mm HEPES-KOH (pH 7.7), 3 mm MgCl_2_, 1 mm DTT, 150 mm KCl, 0.1% Nonidet P-40) and incubated with either monoclonal anti-HA (ab1424, Abcam) or anti-Myc (M5546, Sigma) antibodies or no antibody for 2 h at 4 °C. Crude extract (100 μl) was added to the columns with the immobilized antibodies and incubated for 2 h at 4 °C. Washes were carried out with ice-cold wash buffer (20 mm HEPES-KOH, pH 7.7, 3 mm MgCl_2,_ 150 mm KCl, 0.1% Nonidet P-40). Purified proteins were eluted into boiling SDS-PAGE loading buffer, boiled for 5 min, and centrifuged at 1000 × *g* for 7 min. The supernatant was removed, and 15 μl was loaded onto either 8 or 10% polyacrylamide gels.

##### Flow Cytometry

RNAi was induced in the BF *T. brucei* T3, T3-FYRP, and T3-RCCP cell lines, and cells were harvested at different time points, washed once in PSG buffer, and fixed in 2% paraformaldehyde. These cell lines contain an *eGFP* reporter gene inserted behind the promoter of a silent *VSG221* expression site. Fluorescence of the cells was monitored in the FL-1 channel using a BD FACSCalibur (BD Biosciences). CellQuest software (BD Biosciences) was used to calculate the average of 100,000 events (BD Biosciences). The -fold ES derepression was calculated by dividing the average FL-1 fluorescence of RNAi-induced populations at each time point by the average FL-1 fluorescence of uninduced populations. Three independent experiments were performed with each cell line with the S.D. values shown with *error bars*.

##### Analysis of Nucleic Acids and Proteins

The BF *T. brucei* T3-FYRP and T3-RCCP cell lines were used for quantitative RT-PCR. RNAi was induced against FYRP or RCCP, and total RNA was isolated at various time points using the RNeasy kit (Qiagen). RNA was treated with DNase using the TURBO DNA-free kit (Ambion), and reverse transcription was carried out using the Omniscript RT kit (Qiagen) with random hexamer primers (Promega). qPCR was performed on a 7500 Fast Real-Time PCR system (Applied Biosystems) using Brilliant II SYBR Green (Agilent Technologies). We used primers that our bioinformatic analyses indicated would recognize single copy sequences within the *T. brucei* genome (results not shown), and the reaction conditions for each primer pair were individually optimized. Control reactions without RT were performed using DNase-treated RNA for each time point. Transcript levels were normalized to levels for γ-tubulin transcripts and plotted as -fold increase with respect to the 0 h time point. Three independent experiments were performed, with S.D. values shown with *error bars*.

Whole-cell protein lysates were prepared by washing cells once in PSG buffer, followed by lysis in boiling hot 1× Laemmli buffer at an end concentration of 10^5^ cells/μl. This was incubated at 100 °C for 10 min before loading onto 6 or 10% SDS-polyacrylamide gels. Gels were blotted onto Hybond-P membrane (Amersham Biosciences) and probed with rabbit polyclonal antibodies against Protein A, BiP (gift of Jay Bangs) (64), TbISWI (53), NLP (55), HA tag (ab9106, Abcam), Myc tag (ab9110, Abcam), TDP1 (gift of Klaus Ersfeld) (65), and RCCP. ECL peroxidase-labeled anti-rabbit IgG antibody (GE Healthcare) was used to detect bound antibodies, and the blots were visualized with Western Lightning Plus ECL (PerkinElmer Life Sciences) or ECL Plus (Amersham Biosciences).

##### Chromatin Immunoprecipitation

Chromatin immunoprecipitation (ChIP) was performed as described previously (55). HA-tagged copies of TbISWI, NLP, and FYRP in the BF *T. brucei* 221GP1(VO2+) cell line (60) were immunoprecipitated using a monoclonal anti-HA antibody (ab1424, Abcam) and compared with experiments performed with the parental cell line. RCCP was immunoprecipitated using polyclonal rabbit antiserum raised against RCCP, and as a control, the same amount (20 μl) of rabbit preimmune serum was used. As a negative control, samples where no antibody was used were included for all ChIPs. The ChIP material was analyzed using qPCR, and the final values for the percentage immunoprecipitated were obtained by subtracting the relevant no antibody control from the HA or RCCP ChIP and then dividing by the total input. Three independent experiments were performed for TbISWI, FYRP, and RCCP, and the S.D. values are shown with *error bars*. One representative NLP ChIP was performed and analyzed by qPCR because this has already been investigated (55). Statistical analyses were performed using a one-way analysis of variance followed by a Tukey test to compare pairs of values.

## Results

### 

#### 

##### Identification of TbISWI Partners

**FIGURE 1.**
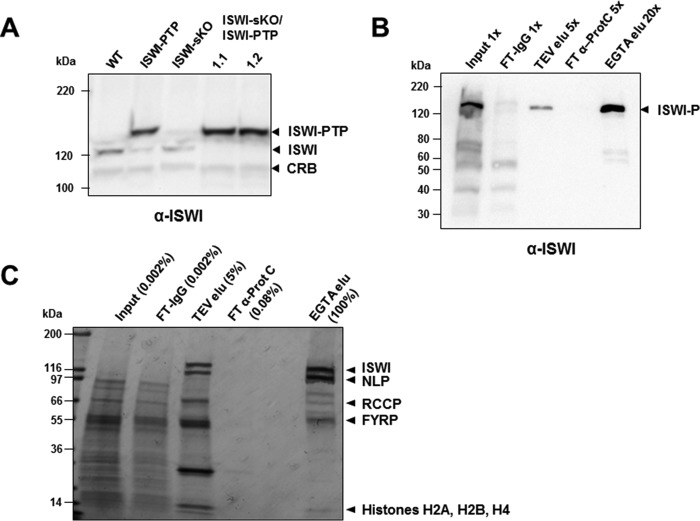
**Identification of ISWI partners in *T. brucei*.** *A*, tagging TbISWI with the PTP epitope. Western blot analysis of whole-cell protein lysates from procyclic form *T. brucei* lines probed with an anti-TbISWI antibody. Extracts from WT cells are compared with those from cells where a single TbISWI allele was either C-terminally tagged with the PTP epitope (ISWI-PTP) or knocked out (ISWI-sKO) or in two clones (1.1 and 1.2) where one TbISWI allele was knocked out and the other allele was PTP-tagged (ISWI-sKO/ISWI-PTP). Relevant bands are indicated, including TbISWI tagged with PTP (ISWI-PTP), untagged ISWI, or a cross-reactive band (*CRB*) which functions as a loading control. The signal for ISWI-PTP is particularly strong, presumably because the tagged protein binds to both the primary and the secondary antibodies. An equivalent of 1 × 10^7^ cells were analyzed on a 6% gel. Size markers in kDa are indicated on the *left. B*, enrichment of PTP-tagged TbISWI using the TAP procedure. Samples isolated during the procedure were monitored using Western blot analysis of a 4–15% SDS-polyacrylamide gel. Samples from the input (1×), the IgG column flow-through (*FT-IgG*), the TEV protease eluate (*TEV elu*) (5×), flow-through from the anti-Protein C column (*FT* α-*ProtC*), or flow-through from the final eluate (*EGTA elu*) (20×) were compared. The blot was probed with an anti-TbISWI antibody. The location of TbISWI-PTP following TEV cleavage (ISWI-P) is indicated with an *arrowhead. C*, monitoring of TbISWI complex purification using Coomassie Blue staining of an SDS-polyacrylamide gel. A sample of the input (0.002% total) was compared with a sample of the IgG column flow-through (*FT-IgG*; 0.002%), the TEV eluate (*TEV elu*; 5% total), flow-through anti-Protein C column (*FT* α-*ProtC*; 0.08%), or the total concentrated EGTA eluate (*EGTA elu*; 100%). Bands were excised for mass spectrometry analysis, and the main hit for each band is indicated *beside* the *arrowhead*. In addition to TbISWI, three TbISWI-interacting partners were identified: the previously identified nucleoplasmin-like protein NLP and two novel proteins that we called RCCP (∼70 kDa) and FYRP (∼54 kDa). Histones H2A, H2B, and H4 (which ISWI interacts with) were also purified from the final eluate. Additional minor bands that are not labeled contained peptides corresponding to TbISWI, NLP, RCCP, and FYRP. Size markers in kDa are indicated on the *left*.

In general, in eukaryotes, ISWI is present in one or more functional complexes (10). We performed BLAST searches of the *T. brucei* genome with the sequences of ISWI partners in other organisms but were unsuccessful in detecting potential homologues in *T. brucei*. In order to identify TbISWI-interacting proteins, we used a TAP tagging approach with a PTP epitope tag (58). We generated a PF *T. brucei* cell line expressing a C-terminal PTP-tagged TbISWI protein from its endogenous locus (Fig. 1*A*). The second allele of TbISWI was knocked out (ISWI-sKO) without resulting in a growth defect, demonstrating that PTP-tagged TbISWI is fully functional (Fig. 1*A*).

This cell line was used for the TAP tagging procedure (Fig. 1, *B* and *C*). Briefly, a crude protein extract was first purified by IgG affinity chromatography, and the TEV protease was used to cleave off the Protein A portion of the PTP tag. Subsequently, the TEV protease eluate underwent anti-Protein C affinity purification, and the final purified products were eluted with EGTA. The concentrated proteins, along with fractions obtained throughout the purification procedure, were separated by SDS-PAGE under denaturing conditions (Fig. 1, *B* and *C*).

We monitored the enrichment of TbISWI-PTP by Western blot using an anti-TbISWI antibody (Fig. 1*B*) to show that the purification was successful. The same samples were separated by SDS-PAGE and stained with Coomassie, where a range of bands were easily detectable in the final eluate (Fig. 1*C*). These bands were excised, and the associated proteins were identified by liquid chromatography/tandem mass spectrometry (LC-MS/MS). As expected, TbISWI-P was recovered in the EGTA eluate (Fig. 1*C*) (108 unique peptide hits) as well as histones H2A, H2B, and H4. Because ISWI is a nucleosome remodeler, this interaction with histones is not unexpected. However, three additional potential TbISWI partners were discovered with molecular masses of ∼110, 70, and 55 kDa, with 70, 32, and 26 unique peptide hits, respectively.

**FIGURE 2.**
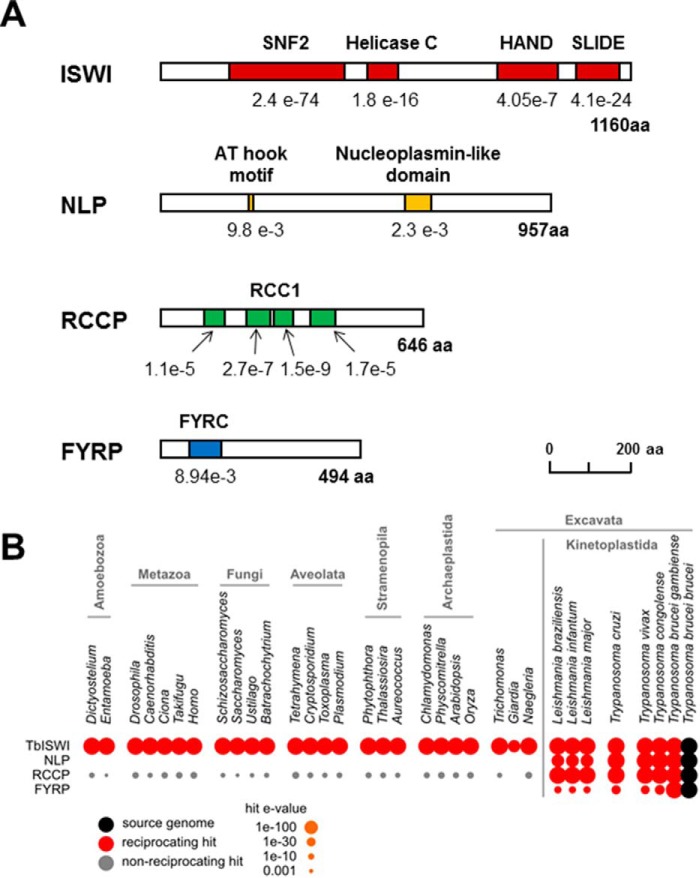
***T. brucei* ISWI and its partners.** *A*, schematics of TbISWI and its partners with relevant protein motifs indicated with *colored boxes*. TbISWI has a SNF2_N domain, a helicase C domain, a HAND, and a SLIDE domain. NLP contains an AT hook motif and a nucleoplasmin-like domain. RCCP contains four RCC1 (regulator of chromosome condensation) domains. FYRP contains a FYRC (F/Y-rich C-terminal) domain. The domains (with *e* values indicated *below*) were identified using Pfam 27, with the exception of NLP, where MyHits was used (*ISB-SIB*), and ISWI, where Superfamily version 1.75 was also used. *B*, conservation of TbISWI components across eukaryotes. Distribution of homologs was investigated by analysis of BLAST hits in the predicted proteomes of model species from a range of eukaryotic lineages. *Spot size* represents the strength of BLAST hit (*e* value). *Red*, reciprocal best-BLAST hits between proteomes (probably orthologs); *gray*, non-reciprocating hits (probably paralogs).

Unexpectedly, one of these TbISWI-interacting proteins (110 kDa) was the NLP, which contains an AT-hook and a nucleoplasmin-like domain and is essential in BF *T. brucei* (Fig. 2*A*) (55). We had previously shown that NLP plays a role in ES silencing, and knockdown of NLP results in 45–65-fold derepression of an *eGFP* reporter gene inserted immediately downstream of a silent ES promoter (55). Additionally, two TbISWI-interacting proteins that had not been previously characterized were identified. We named the 70-kDa protein RCCP (Tb927.11.10330) because it has four RCC1 (regulator of chromosome condensation 1)-like domains (Fig. 2*A*). The human RCC1 protein is a cell cycle regulator that binds chromatin and acts as a guanine nucleotide exchange factor for Ran GTPase (66).

The 54-kDa TbISWI-interacting protein was named FYRP (Tb927.7.1060) because it has an N-terminal phenylalanine/tyrosine-rich (FYR) domain (Fig. 2*A*). FYR domains are poorly characterized but have been found in chromatin-associated proteins, including histone methyltransferases, such as trithorax (67, 68). Because NLP was one of the TbISWI-interacting proteins, we subsequently also performed tandem affinity purification with NLP tagged with the PTP epitope (results not shown). Again, we identified TbISWI, RCCP, and FYRP with a significant number of unique peptide hits (95, 46, and 26, respectively).

We next investigated whether TbISWI and its interacting partners were conserved in a range of different eukaryotic species (Fig. 2*B*). TbISWI is orthologous to ISWI in *S. cerevisiae* and SMARCA1/SNF2L in *Homo sapiens*. This protein is very highly conserved across eukaryotes, with orthologs present in all species analyzed from a wide range of lineages. In contrast, TbISWI-interacting proteins appear to be restricted to the Kinetoplastida. NLP and FYRP homologs are only identifiable in this lineage. RCCP is an RCC1 repeat domain-containing protein, of which there are several members in trypanosomes and most other eukaryotes. However, the RCCP paralog itself is specific to the Kinetoplastida (Fig. 2*B*).

##### TbISWI Interacts with Its Partners Forming the TbISWI Complex (TbIC) in T. brucei

**FIGURE 3.**
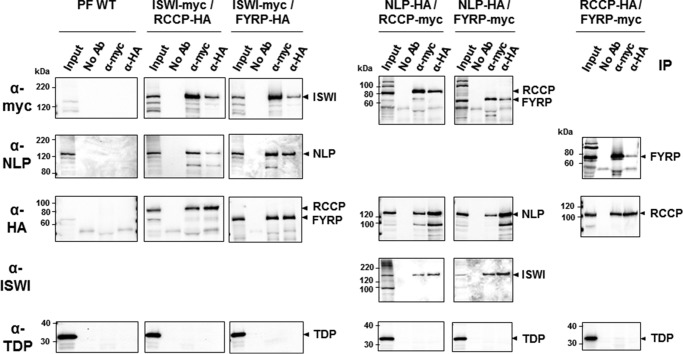
**Co-immunoprecipitation reactions show interaction between *T. brucei* ISWI and each of its partners in insect form trypanosomes.** *T. brucei* cell lines were generated using procyclic form trypanosomes (*PF WT*) containing a Myc-tagged TbISWI and either an HA-tagged RCCP or HA-tagged FYRP (ISWI-Myc/RCCP-HA or ISWI-Myc/FYRP-HA, respectively). Alternatively, cell lines contained HA-tagged NLP and either Myc-tagged RCCP or Myc-tagged FYRP. Last, cell lines containing HA-tagged RCCP and Myc-tagged FYRP were analyzed. Using protein lysates from these cell lines, immunoprecipitation reactions were performed using either anti-Myc (α-*myc*) or anti-HA (α-*HA*) monoclonal antibodies or a no antibody control (*No Ab*). These immunoprecipitated samples were separated on SDS-polyacrylamide gels together with samples from the input (0.4% amount used for immunoprecipitation). Each blot was probed with antibodies against Myc (α-*myc*), NLP (α-*NLP*), HA (α-*HA*), TbISWI (α-*ISWI*), or the chromatin protein TDP1 (α-*TDP*), which served as a negative control. Relevant proteins are indicated on the *right* with *arrowheads*. Protein size markers in kDa are indicated on the *left*.

Is ISWI present in one or multiple complexes in *T. brucei*? Typically, in different eukaryotes, ISWI is a component of a number of functional ISWI complexes, with discrete roles depending on the composition of the subunits (8, 10). We investigated whether the potential TbISWI partners identified through TAP affinity purification were indeed interacting with TbISWI and with each other. We performed co-IP experiments in PF cells that contained Myc-tagged TbISWI and HA-tagged RCCP or FYRP proteins. Immunoprecipitation with either anti-Myc or anti-HA monoclonal antibodies was followed by Western blot analysis to determine whether other potential TbISWI complex components were co-purified. (Fig. 3). We used an anti-Myc antibody to detect TbISWI-Myc (138 kDa), polyclonal anti-NLP antibody to detect NLP (107 kDa), and anti-HA antibody to detect RCCP-HA and FYRP-HA (74 and 57 kDa, respectively). NLP, RCCP, and FYRP were all co-purified when TbISWI was pulled down. We also found that TbISWI and NLP co-purified when RCCP or FYRP were immunoprecipitated.

**FIGURE 4.**
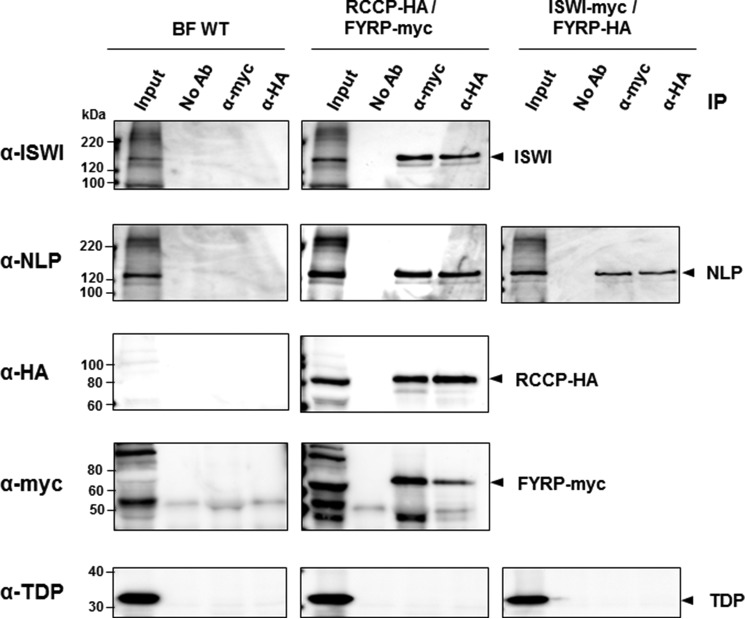
**Co-immunoprecipitation experiments show that TbISWI partners show similar interactions in bloodstream form as well as procyclic form *T. brucei*.** Lysates from bloodstream form *T. brucei* 221GP1(VO2+) (*BF WT*) containing RCCP tagged with the HA epitope and FYRP tagged with the Myc epitope (*RCCP-HA/FYRP-myc*) were compared with a line containing TbISWI tagged with the Myc epitope and FYRP tagged with the HA epitope (*ISWI-myc/FYRP-HA*). Immunoprecipitation experiments were carried out with antibodies against the Myc (α-*myc*) or HA epitope (α-*HA*) or a no antibody control (*No Ab*). These immunoprecipitated samples were separated on SDS-polyacrylamide gels together with samples from the input (0.4% of the amount used for immunoprecipitation). Blots were probed with antibodies against TbISWI, NLP, HA, Myc, or the chromatin protein TDP as a negative control. Relevant proteins are indicated on the *right* with *arrowheads*. Sizes of protein markers are indicated on the *left* in kDa.

Further co-IP experiments were performed in PF cells with different combinations of tagged proteins, and it was shown that when FYRP is pulled down, RCCP is co-purified, and *vice versa* (Fig. 3). Co-IP experiments showed similar interactions between TbISWI and its proposed partners in BF cells (Fig. 4). These extensive co-IP experiments argue that there is at least one ISWI complex containing TbISWI, NLP, RCCP, and FYRP and that all members of this complex interact with each other in both BF and PF life cycle stages of *T. brucei*.

**FIGURE 5.**
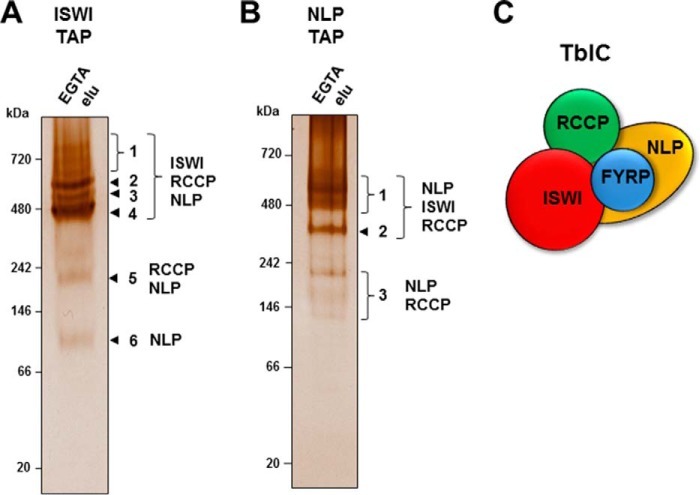
**Native gels show the presence of a single predominant TbISWI complex in *T. brucei*.** *A*, TAP affinity purification was performed with lysates from procyclic form *T. brucei* containing TbISWI tagged with the PTP epitope. The purified material was separated on a 4–15% non-denaturing gel and silver-stained. The bands that were excised and sent for mass spectrometry are indicated with *numbers* on the *right*. The proteins subsequently identified in each band are also indicated. The sizes of the native gel protein marker are indicated in kDa on the *left. B*, as in *A* except that lysates were used from procyclic form cells where the TbISWI partner NLP was tagged with the PTP epitope. *C*, a schematic of the *T. brucei* TbISWI complex (TbIC) with TbISWI and its different partners indicated with *colored spheres*.

To elucidate whether TbISWI forms one complex or multiple subcomplexes, TbISWI-PTP and its co-purified components from the TAP affinity purification experiments were separated under nondenaturing conditions and silver-stained (Fig. 5*A*). Similarly, the same experiment was performed with TAP affinity-purified NLP-PTP (Fig. 5*B*). The visible bands were excised and analyzed by mass spectrometry. A predominant major band was seen in both cases, corresponding to either TbISWI or NLP complexed with each other and with RCCP.

FYRP was detected in both experiments, albeit below the threshold score of 80, indicating weak association with this complex. However, based on its score in the initial TbISWI and NLP TAP tagging experiments and the extensive co-IP experiments, we are confident that FYRP is a true member of the TbISWI complex. Additional minor bands observed below the main band contain different stoichiometries of complex partners indicating possible different degradation states of a single complex. These data therefore indicate that there is a single major *T. brucei* ISWI complex (TbIC) (Fig. 5*C*). However, we cannot exclude the presence of additional minor subcomplexes composed of just some of the TbISWI complex subunits.

##### Depletion of FYRP or RCCP Results in Derepression of Silent VSG Expression Sites

**FIGURE 6.**
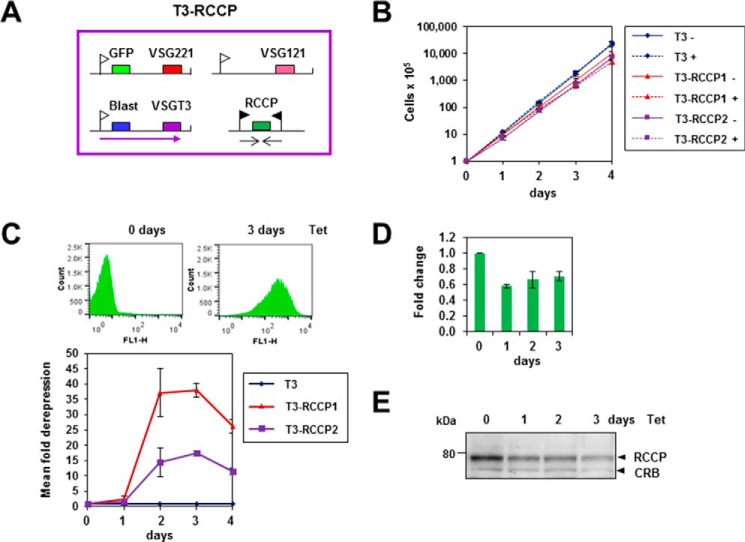
**Knockdown of RCCP in bloodstream form *T. brucei* leads to *VSG* expression site derepression**. *A*, schematic of the *T. brucei* T3-RCCP cell line containing a silent GFP gene in the *VSG221* expression site and an active *VSGT3* expression site containing a blasticidin resistance gene (*blast*). RNAi against RCCP can be expressed from opposing tetracycline-inducible T7 promoters. Relevant genes are indicated with *colored boxes*, and transcription is indicated with *arrows*, with expression site promoters shown with *white flags* and T7 promoters with *black flags. B*, there is no significant reduction in growth after knockdown of RCCP. The parental T3 cell line and the T3-RCCP1 and T3-RCCP2 clones were incubated in the presence (+) or absence (−) of tetracycline. The cumulative growth was plotted over time, with *error bars* indicating the S.D. from three replicate experiments. *C*, depletion of RCCP leads to 17–37-fold derepression of the silent *VSG221* expression site, as monitored using GFP. The *top panel* shows representative flow cytometry traces in the FL-1 channel either before or after induction of RCCP RNAi with tetracycline for 3 days. The *bottom panel* shows the mean -fold derepression in the T3-RCCP1 or T3-RCCP2 clones compared with the parental cell line (T3) after the induction of RCCP1 RNAi for the time indicated in days. *Error bars*, S.D. from three independent experiments. *D*, knockdown of RCCP transcript after the induction of RCCP RNAi for the time indicated in days. Transcript levels were determined using quantitative RT-PCR, normalized using γ-tubulin, and are shown relative to the 0 h time point. The results shown are the average of three independent experiments with *error bars* showing S.D. *E*, reduction in levels of RCCP protein after the induction of RCCP RNAi with tetracycline (*Tet*) for the time indicated in days. Protein lysates from the T3-RCCP cell line in the presence of RCCP RNAi were analyzed by Western blot. Blots were probed with a rabbit polyclonal antibody against RCCP. A cross-reactive band (*CRB*) is indicated as a loading control. The size of a marker protein in kDa is indicated on the *left*.

We have previously established that both TbISWI and NLP play a role in ES silencing (53, 55). We investigated the role of RCCP and FYRP on ES control using a BF *T. brucei* VSGT3-expressing reporter cell line where eGFP had been inserted immediately downstream of the promoter of the inactive *VSG221* ES (53). RNAi was induced against RCCP, resulting in a reduction in transcript levels to about 60% of normal levels, with a simultaneous reduction in levels of protein (Fig. 6). Only a minor reduction in cell growth was observed. However, there was an observed 17–37-fold derepression of *eGFP* in the silent *VSG221* ES after 72 h as monitored in the FL-1 channel using flow cytometry (Fig. 6*C*).

**FIGURE 7.**
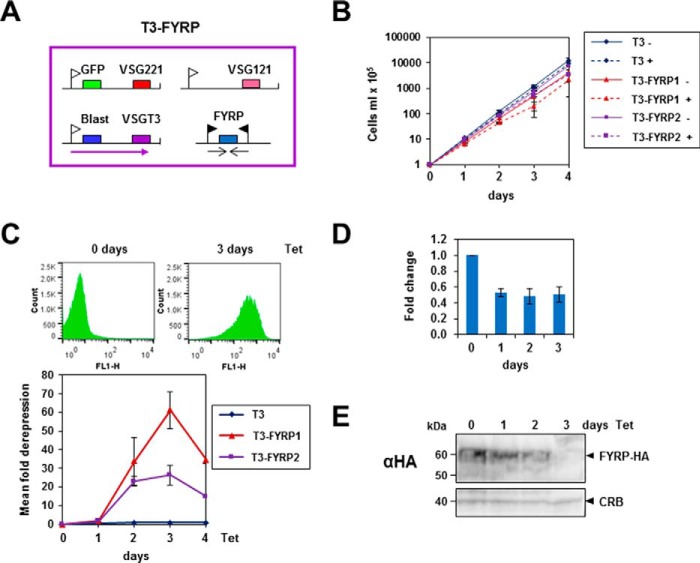
**Knockdown of FYRP in bloodstream form *T. brucei* leads to derepression of the silent *VSG221* expression site.** *A*, the schematic shows the *T. brucei* T3-FYRP reporter cell line, which has an active *VSGT3* expression site containing a blasticidin resistance gene (*blast*) and an inactive *VSG221* expression site with a silent GFP gene. RNAi against FYRP can be expressed from a construct containing two opposing tetracycline-inducible promoters. Relevant genes are shown with *colored boxes*, transcription is indicated with *arrows*, expression site promoters are shown with *white flags*, and T7 promoters are shown with *black flags. B*, only minor growth reduction is observed after the induction of FYRP knockdown. The parental T3 cell line, as well as the T3-FYRP1 and T3-FYRP2 clones, was incubated in the presence (+) or absence (−) of tetracycline for the time indicated in hours. The cumulative growth was plotted against time, with *error bars* indicating the S.D. of three replicate experiments. *C*, FYRP knockdown leads to 26–61-fold derepression of the silent *VSG221* expression site as monitored using GFP. The *top panel* shows representative flow cytometry histograms in the FL-1 channel either before or after induction of FYRP RNAi for 72 h using tetracycline (*Tet*). The *bottom panel* shows mean derepression in the *T. brucei* T3-FYRP1 or T3-FYRP2 clones compared with the parental cell line (T3) after induction of FYRP RNAi for the time indicated in hours. *Error bars*, S.D. from three independent experiments. *D*, decrease in levels of FYRP transcript in *T. brucei* T3-FYRP1 following the induction of FYRP RNAi. Transcript levels were determined using quantitative RT-PCR, normalized to γ-tubulin, and shown relative to values at the 0 h time point. Results are the average of three independent experiments, with *error bars* showing the S.D. *E*, efficient knockdown of FYRP protein after the induction of FYRP RNAi. Protein lysates were analyzed after the induction of FYRP RNAi with tetracycline for the time indicated in hours in a cell line containing a copy of FYRP tagged with the HA epitope. Western blot analysis was performed using an anti-HA antibody (α*HA*). A cross-reacting band (*CRB*) is indicated *below* as a loading control. The sizes of protein markers in kDa are indicated on the *left*.

We performed a similar analysis of the role of FYRP (Fig. 7). The FYRP transcript was reduced to 50% of normal levels after 24 h. FYRP protein knockdown was investigated using a cell line with an HA-tagged copy of FYRP, which was knocked down to undetectable levels after a 96-h induction of RNAi (Fig. 7*E*). Here too, although the induction of RNAi resulted in only a minor reduction in cell growth (Fig. 7*B*), there was 26–61-fold derepression of the silent *VSG221* ES.

##### Genomic Localization of the TbISWI Complex

The native gels and the co-IP experiments suggested that there is a single predominant TbISWI complex (TbIC) in *T. brucei*. However, to investigate this further, we determined the genomic localization of the four potential components using ChIP experiments. ChIP was performed in different BF cell lines expressing either HA-tagged TbISWI, HA-NLP, or HA-FYRP, using a monoclonal anti-HA antibody. Multiple attempts of ChIP using HA epitope-tagged RCCP proved unsuccessful, indicating a possible lack of accessibility of the HA epitope to antibodies when the ISWI complex is in association with DNA. We therefore used a rabbit polyclonal antibody against RCCP in the RCCP ChIP experiments.

**FIGURE 8.**
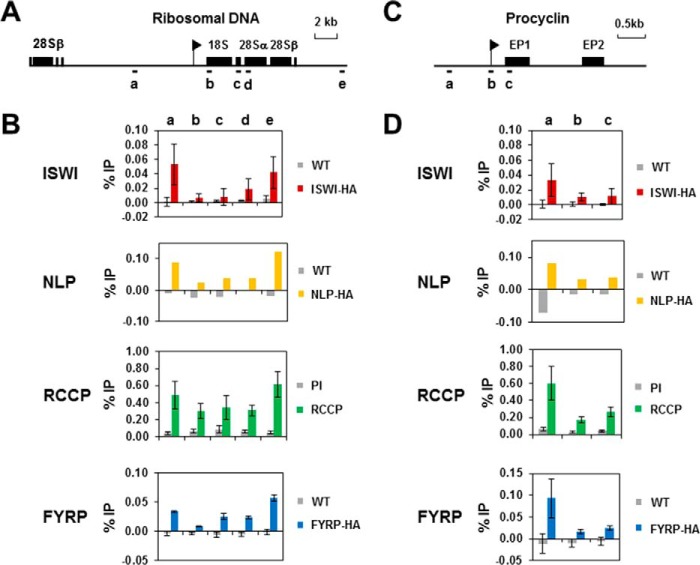
***T. brucei* ISWI and its partners colocalize at the Pol I-transcribed rDNA and procyclin loci in bloodstream form *T. brucei*.** *A*, schematic of a typical rDNA transcription unit, with genes indicated with *black boxes*, and the rDNA promoter indicated with a *black flag*. Regions analyzed by qPCR are indicated with *letters. B*, colocalization of TbISWI and its partners at the rDNA locus. Chromatin from *T. brucei* ISWI-HA, NLP-HA, FYRP-HA, or WT cells was immunoprecipitated with an anti-HA antibody. Chromatin from parental cells was immunoprecipitated with an anti-RCCP antibody, and rabbit preimmune serum (*PI*) was used as a negative control. The genomic regions analyzed are indicated in the *schematic* and listed *above* the *graphs*. Results are presented as the amount immunoprecipitated (percentage of input (% *IP*)) after subtraction of the no antibody control. Results shown are the mean of three independent experiments with the S.D. indicated with *error bars*, apart from NLP. Here the results are from one representative ChIP experiment because similar data have been published previously by Narayanan *et al.* (55). *C*, a diagram of the EP procyclin locus transcribed by multifunctional Pol I. A *black flag* depicts the procyclin promoter, and *letters* indicate the regions that were analyzed using qPCR. *D*, different TbISWI partners colocalize at the procyclin locus. Immunoprecipitated chromatin at the procyclin genomic loci was analyzed as indicated in the legend for *B*. Regions analyzed are shown *above* the *graphs*.

We first investigated the localization of the TbISWI complex components at the RNA Pol I-transcribed rDNA loci (Fig. 8*A*). TbISWI and NLP are relatively depleted within Pol I transcription units but enriched at non-transcribed regions (53, 55). This pattern of localization was also observed for RCCP and FYRP (Fig. 8*B*). In the case of FYRP, the statistical significance of this differential localization was extremely significant (*p* < 0.001) (primer pairs a *versus* primer pairs b or primer pairs e *versus* primer pairs b, c, or d). In the case of RCCP, although there was a trend, this was not statistically significant. Similarly, at the Pol I-transcribed procyclin loci (Fig. 8*C*), TbISWI and NLP are relatively enriched upstream compared with within the transcription units (53, 55). This was also the case for both RCCP and FYRP with a statistical significance of *p* = 0.01–0.05 (primer pairs a *versus* primer pairs b or c) in both cases (Fig. 8*D*).

Pol II transcription units in *T. brucei* are polycistronic. Pol II transcription initiates in SSRs, where two opposing transcription units diverge, and terminates where they converge. TbISWI was proposed to be enriched at these SSRs and particularly in the regions around divergent SSRs containing promoters (54). ChIP experiments with ISWI are very difficult to perform, presumably as a consequence of the relatively low affinity of this chromatin remodeler for DNA.

**FIGURE 9.**
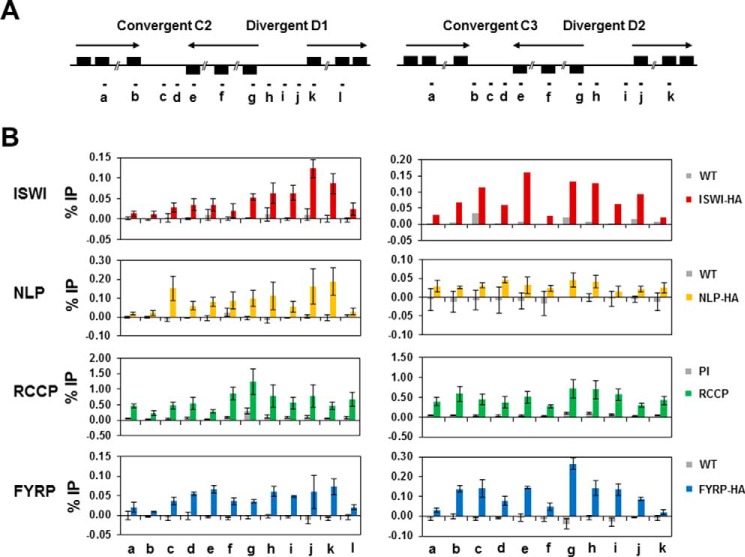
**Location of different TbISWI partners at two different Pol II convergent and divergent strand switch regions.** *A*, schematic of different Pol II SSRs from chromosome 10 (convergent regions C2 and C3 and divergent regions D1 and D2). These regions were initially described by Siegel *et al.* (35), and also analyzed by Stanne *et al.* (54). Convergent SSRs contain putative Pol II termination sites, and divergent SSRs contain putative Pol II promoters. Genes are indicated with *black boxes*, with *arrows* showing the direction of transcription. Genomic regions analyzed by qPCR are indicated with *letters*. Primer pairs a, f, and l are located approximately in the middle of the polycistronic transcription units. *B*, distribution of the TbISWI partners at different Pol II SSRs. ChIP was performed using an anti-HA antibody on either WT cells or cells containing an HA epitope-tagged ISWI, HA-NLP, or HA-FYRP. An anti-RCCP antibody was used to immunoprecipitate RCCP and is compared with ChIP performed with rabbit preimmune (*PI*) serum. The results are expressed as percentage of total input (% *IP*), followed by subtraction of the no antibody control. Results are shown as the average of three independent experiments, with *error bars* showing the S.D. with the exception of some of the ISWI results because these confirm previously published data (54).

TbISWI, NLP, RCCP, and FYRP appeared to bind regions around different Pol II SSRs (Fig. 9). In parallel, ChIP experiments were also performed with histone H3, serving as a positive control for the ChIP procedure (result not shown). There was possible colocalization of ISWI subunits at the SSR divergent regions D1 and D2; however, these results were not statistically significant. All members of the TbISWI complex associate with chromatin and show a trend of localizing to similar genomic regions, which is statistically significant at Pol I loci. All of these different experimental approaches that show all TbIC components interacting and present at a variety of genomic loci argue that there is a single predominant ISWI complex in *T. brucei.*

## Discussion

In eukaryotes, the ISWI chromatin remodeler is typically present in a variety of different complexes with distinct functions, depending on exactly which subunits ISWI is partnered up with. Here, we have characterized TbISWI and its interacting partners in *T. brucei* and provide evidence for a single major ISWI complex (TbIC) in both BF and PF *T. brucei*. Using a number of different experimental methods, we show that all of the TbIC subunits are expressed and interact with each other in both trypanosome life cycle stages. The previously characterized nucleoplasmin-like protein NLP was found to be a member of this TbIC complex. This unexpected discovery explains the observation that knockdown of either TbISWI or NLP leads to similar phenotypes, including the derepression of *VSG* ESs. In addition, using TAP affinity purification with either TbISWI or NLP, we identify two novel and previously uncharacterized TbIC components: RCCP and FYRP. Neither of these ISWI partners is a homologue of known ISWI partners in other eukaryotes. However, both proteins contain amino acid sequence motifs indicating a possible interaction with chromatin.

The TbISWI-interacting RCCP protein contains four RCC1 protein motifs, which characterize the RCC1 superfamily of proteins (66). The RCC1 family is a diverse group of proteins which contain variable numbers of RCC1-like domains, with a tertiary structure resembling a seven-bladed propeller (69). RCC1 is the best characterized member of this family and is a DNA-binding protein that regulates the onset of chromosome condensation (70). RCC1 is localized to chromatin throughout the cell cycle and is a guanine nucleotide exchange factor for Ran (71–73). RCC1 binds nucleosomes, recruits Ran to the chromatin, and activates Ran nucleotide exchange activity (72, 74). It therefore plays a central role in establishing the RanGTP concentration gradient around the chromosome, which is key for a number of processes to occur, including mitosis (75–77). In this regard, it is interesting that it has been reported that in *Xenopus*, ISWI is a RanGTP-dependent microtubule-associated protein required for chromosome segregation (78). Although in *T. brucei*, knockdown of TbISWI and its subunits leads to derepression of *VSG* ESs, we have not seen obvious disruption of chromosome segregation.

In contrast, the TbISWI-interacting protein FYRP is characterized by a FYRC domain. FYRC protein motifs contain a phenylalanine- and tyrosine-rich region that is poorly characterized and is found in an assortment of chromatin-associated proteins (68). FYRC domains are typically found in association with protein modules that recognize histone modifications (79). FYRC motifs have been identified in the *Drosophila* trithorax protein, involved in the epigenetic regulation of gene expression during fly development, and X chromosome-interacting proteins (67).

One possibility that could explain our data is that in *T. brucei*, FYRP is the most prone to disassociate from the TbIC ISWI complex compared with the other three subunits. Although we repeatedly identified FYRP by mass spectrometry using TAP affinity purification with either ISWI or NLP as bait, the score was consistently the lowest of the four TbIC components. In addition, FYRP was not identified in the TbIC complex using native gel analysis. However, co-IP experiments showed clear interaction of FYRP with every other TbIC subunit (TbISWI, NLP, and RCCP). In addition, ChIP experiments showed a trend for localization of FYRP with other TbIC members on similar regions of genomic DNA. Similarly, knockdown of FYRP also led to comparable derepression of silent *VSG* ESs as observed after knockdown of the other TbIC subunits. Our data therefore indicate that FYRP could have a weak or transient interaction with other complex members, making it prone to disassociation during protein affinity purification.

Is there indeed only one ISWI complex in *T. brucei*? Both the TbISWI and NLP affinity purification experiments identified each other as well as the RCCP and FYRP subunits. In addition, as mentioned above, co-IP experiments in both life cycle stages show that all four components interact with each other, and ChIP experiments indicate that all four proteins associate with similar regions of genomic DNA. Therefore, all of the available evidence, using a variety of different experimental approaches, would argue that a single predominant TbISWI complex is present in the early branching eukaryote *T. brucei*. As expected for subunits participating in the same complex, knockdown of each of these TbIC subunits leads to *VSG* ES derepression. However, these experiments do not rule out the presence of minor TbISWI complexes containing a subset of the subunits.

Chromatin remodelers, including ISWI complexes, are extremely difficult to analyze using ChIP (80). This may be indicative of the transient nature of the interactions between these remodeling complexes and specific DNA sequences as they move along the genome changing nucleosome spacing (81). Despite these technical hurdles, colocalization of ISWI with different interacting subunits using ChIP can indicate the presence of discrete functional ISWI complexes at different genomic locations (80). Previous ChIP analyses of TbISWI have argued that there is a possible enrichment of TbISWI at the Pol II SSRs, which contain transcriptional boundaries, including Pol II promoters and terminators (35, 54). This is comparable with what has been found in other organisms, including *S. cerevisiae*.

In *S. cerevisiae*, ISWI is important for regulation of Pol II transcription, and ISWI variants are found both within Pol II gene bodies and at both promoters and terminators. The Isw1 variant has different functions, depending on which Ioc subunits it is partnered up with (14, 17). Isw1 in complex with Ioc3 forms the Isw1a complex, which represses initiation of transcription at Pol II promoters (15). In contrast, Isw1 partnered up with the Ioc2 and Ioc3 subunits forms the Isw1b complex, which either acts within Pol II coding regions to control elongation of transcription or alternatively facilitates transcription termination (15). The Isw2 ISWI variant is particularly enriched at the nucleosome-depleted region around Pol II promoters, where it appears to play a role in maintaining a high density of nucleosomes within the Pol II-transcribed gene bodies (81). This reduces the amount of inappropriate Pol II transcription initiation from gene internal cryptic sites and suppresses antisense transcription.

In *T. brucei*, we found a trend for TbISWI and the NLP, RCCP, and FYRP subunits binding at both divergent and convergent Pol II strand switch regions; however, these data supporting four proteins being relatively enriched in these regions were not statistically significant. This relative simplicity of ISWI complex architecture could be a consequence of the lack of control of Pol II expression in *T. brucei* at the level of either transcription initiation or elongation (82).

In most eukaryotes, Pol I exclusively transcribes the rDNA arrays, of which typically about half are transcriptionally silent (83). ISWI variants also play a role in this regulation of Pol I, which in mammals is mediated by the ISWI-containing NoRC complex consisting of ISWI (SNF2H) in complex with the TIP5 subunit (84). This NoRC complex mediates the formation of heterochromatin both at the silent rDNA repeats and at the centromeres (31). In *T. brucei*, all of the TbIC components are located at the rDNA, particularly in the non-transcribed spacers. This is also the case at the Pol I-transcribed procyclin loci and the ESs (85), although no particular enrichment was observed at either active or silent ESs (54). Because knockdown of all of the TbIC components leads to derepression of silent ESs, it is clear that ISWI plays a role in regulation of Pol I transcription in *T. brucei.*

All of our experimental evidence therefore points to a single ISWI-containing complex in *T. brucei*, which is a very early branching eukaryote, although we cannot rule out the presence of relatively minor subcomplexes. The apparent presence of all TbIC components at a range of different genomic loci, including Pol II SSRs, as well as at different Pol I loci argues that the predominant TbIC complex could be multifunctional. Chromatin remodeling enzymes appear to have arisen soon after the origin of the eukaryotic lineage, and as eukaryotic genomes expanded in size and complexity, there was an increasing need for a larger array of specialized chromatin remodeling factors (1). In common with other parasites, *T. brucei* appears to have a relatively reduced set of these chromatin remodelers, coupled with a greatly reduced set of Pol II transcription factors (1, 86). Possibly, as *T. brucei* evolved, large amounts of gene loss occurred as a consequence of the lack of the need for intricate control systems as the organism relied on constitutive transcription by Pol II. We show that the major *T. brucei* TbIC complex contains novel subunits compared with other non kinetoplastid eukaryotes.

The challenge for us now is to understand the role of these unique chromatin remodelers in the maintenance of genome architecture in these ancient eukaryotes. In addition, hopefully, increased knowledge of the role that these divergent chromatin remodelers play in transcriptional control, including that of the VSG expression sites, will allow us to disrupt this process, thereby leading to new forms of antiparasitic therapies.

## Author Contributions

T. S., M. N., A. L., and G. R. designed the study. G. R. and S. R. wrote the paper. B. W. performed bioinformatic analyses. T. S., M. N., S. R., A. L., K. W., M. K., S. W., and J. W. performed the experiments. All authors analyzed the results and approved the final version of the manuscript.

## Footnotes

4 The abbreviations used are:

Pol: polymerase
RCCP: regulator of chromosome condensation 1-like protein
FYRP: phenylalanine/tyrosine-rich protein
VSG: variant surface glycoprotein
ES: expression site
SSR: strand switch region
BF: bloodstream form
PF: procyclic form
TAP: tandem affinity purification
TEV: tobacco etch virus
PTP: Protein C-TEV protease site-Protein A
IP: immunoprecipitation
qPCR: quantitative PCR
NLP: nucleoplasmin-like protein.
